# Impact of Depression on Health-Related Quality of Life in Ulcerative Colitis Patients—Are We Doing Enough? A Single Tertiary Center Experience

**DOI:** 10.3390/life15040612

**Published:** 2025-04-06

**Authors:** Dunja Jaksic, Sasa Vuksanovic, Aleksandar Toplicanin, Jelena Spiric-Milovancevic, Gorica Maric, Aleksandra Sokic-Milutinovic

**Affiliations:** 1 Department of Gastroenterology and Hepatology, Clinic for Internal Medicine, Clinical Hospital Center Zemun, 11 000 Belgrade, Serbia; 2 Clinic for Gastroenterology and Hepatology, University Clinical Center of Serbia, 11000 Belgrade, Serbia; sashavuksanovic9@gmail.com (S.V.); aleksandartoplicanin4@gmail.com (A.T.); spiricjelena77@gmail.com (J.S.-M.); asokicmilutinovic@gmail.com (A.S.-M.); 3 Institute of Epidemiology Faculty of Medicine, University of Belgrade, 11000 Belgrade, Serbia; goricamaric87@gmail.com; 4 Faculty of Medicine, University of Belgrade, 11000 Belgrade, Serbia

**Keywords:** ulcerative colitis, depression, anxiety, alexithymia, health-related quality of life, remission

## Abstract

Ulcerative colitis (UC) significantly impacts patients’ health-related quality of life (HRQOL). This study aimed to evaluate HRQOL and the factors affecting it, and the prevalence of anxiety, depression and alexythimia in patients with UC. This cross-sectional study included 248 UC patients (21 with proctitis, 63 with left-sided UC and 164 with extensive colitis). Data were collected using standardized self-administered questionnaires [a socio-demographic questionnaire, General Anxiety Disorder-7 (GAD-7), the Patient Health Questionnaire-9 (PHQ-9), the Toronto Alexithymia Scale (TAS-20) and the Short Inflammatory Bowel Disease Questionnaire (SIBDQ)]. Clinical data on remission status, extraintestinal manifestations, comorbidities and the use of advanced therapies were also collected. Hierarchical regression analysis of variables predicting SIBDQ score was done. Clinical and laboratory remission was present in 95.6% of the patients. The prevalences of depression, anxiety and alexithymia were 44.7%, 34.3% and 30.2%, respectively. There were no differences in the PHQ-9, GAD-7 and TAS-20 scores in relation to remission status. The average SIBDQ score was 56.5. The patients in remission reported better SIBDQ scores compared to the symptomatic patients (*p* = 0.002). The hierarchical regression analysis showed that remission of disease and a higher depression score influenced HRQOL in the UC patients. The prevalence of depression, anxiety and alexithymia in the UC patients was high. Remission of disease and a high depression score were the main factors related to HRQOL.

## 1. Introduction

Chronic inflammatory bowel diseases (IBD)—ulcerative colitis (UC) and Crohn’s disease (CD)—are characterized by chronic gastrointestinal inflammation and a relapsing–remitting course [1]. The exact pathophysiology of IBD remains unclear. It is understood as a multifactorial interplay involving disturbance in gastrointestinal physiology, intestinal microbiome, immunology, and genetics [2]. At the beginning of the 21st century, the global incidence and prevalence of IBD were rising. The prevalence is rising in Western Europe, North America and Oceania, while the incidence is increasing in Latin America, Asia and Africa [3]. UC is characterized by diffuse inflammation affecting the rectal mucosa and extending to proximal segments of the colon. UC most commonly presents with diarrhea, which may contain blood and mucus. Fever and abdominal pain may also occur, as well as extraintestinal manifestations. The therapeutic options for UC patients include mesalazine, corticosteroids, immunosuppressants, advanced therapies (monoclonal antibodies and small molecules) and surgery.

Health-related quality of life (HRQOL) comprises the aspects of well-being that are related to or affected by the presence of disease or its treatment, as perceived by the patient [4]. A diagnosis of an IBD affects the HRQOL of patients due to their chronic symptoms, early-life onset, unpredictable course, the severity of the symptoms, as well as the side effects of conservative and surgical treatments, which interfere with daily activities and alter the patient’s lifestyle [5]. Short-term and long-term therapeutic goals in UC have evolved from initially controlling the symptoms to healing the mucosa and restoring a normal quality of life. The STRIDE II recommendations, issued by the International Organization for IBD in 2021, state that the ultimate treatment goal is to maintain a normal HRQOL and prevent disability [6].

Psychological symptoms may worsen the HRQOL in outpatients with an IBD [7,8]. The incidence and prevalence of depression and anxiety are higher in IBD patients compared with populations without an IBD [9]. Based on a systematic review of 48 studies, the prevalence of anxiety disorders ranges from 3.8% to 25% in the general population, while in patients with chronic diseases, it is estimated to be as high as 70% [10]. The average prevalence of depression in Europe is 7%, which varies between regions [11]. In patients with an IBD, the prevalence of anxiety is 20.5% and the prevalence of depression is 21.2%, which are higher than those of healthy controls [12,13]. A systematic review and meta-analysis by Barberio et al., which included 30,118 patients, showed that one-third of IBD patients are anxious and a quarter are depressed. During the active phase of the disease, one in two patients is either anxious or depressed [14].

A meta-analysis by Bisgaard et al. revealed that all IBD patients, both adult and pediatric, are at a 1.5-times higher risk of anxiety and depression [15].

The relationship between IBDs and depression and anxiety seems to be bidirectional [15]. The mechanisms behind this relationship include increased levels of pro-inflammatory cytokines, vagal nerve signaling, gut dysbiosis, and changes in brain signaling and morphology [16]. The intestinal microbiota has recently been recognized as an integral part of the brain–gut axis, which can potentially modulate behavioral and cognitive functioning through endocrine, neural and metabolic signaling. This led to a change in terminology and some authors now refer to it as brain–gut–microbiota axis [17,18,19]. Patients who experience depression or anxiety tend to report more frequent disease relapses and a worse general well-being, as the emotional burden exacerbates their physical symptoms [20,21,22,23]. Relapses may worsen anxiety and depression and can make it harder for patients to adhere to treatment regimens or to seek care. 

Certain researchers consider IBDs as examples of a psychosomatic disorder [24]. Alexithymia (“no words for feelings”) is a personality feature characterized by difficulties in identifying, analyzing and verbalizing feelings, restricted imaginal capacities and a limited emotional experience [25]. Alexithymia is considered to be a risk factor for various psychiatric and psychosomatic disorders and its presence positively correlates with anxiety and depression in patients with an IBD [26,27,28]. The co-occurrence of depression, anxiety and alexithymia in IBD patients often creates a vicious cycle.

Patients with UC should be screened for co-existing anxiety and depressive disorders, and, once diagnosed, the patients should be provided with resources to address these conditions [29]. Despite the known impact of these mental health conditions on patients’ morbidity and quality of life, they often go unrecognized and untreated. Addressing this gap requires increased awareness and routine mental health screening to improve the overall outcomes for IBD patients.

This study aimed to evaluate HRQOL using the SIBDQ and the possible factors affecting it, and the prevalence of anxiety, depression and alexithymia in patients with UC. The majority of the previously published studies focused on the prevalence of anxiety, depression and alexithymia in all IBD patients and the data focusing solely on UC patients are limited.

## 2. Materials and Methods

### 2.1. Study Design and Settings

This cross-sectional study was conducted at the Clinic for Gastroenterology and Hepatology, University Clinical Center of Serbia, in Belgrade, from January 2023 to February 2024. All participants gave written informed consent before participating in this study. The study was conducted in accordance with the 1964 Declaration of Helsinki and its later amendments and all applicable Serbian laws and guidelines. The study was approved by the Ethics Committee of the Faculty of Medicine, University of Belgrade (approval number: 17/I-12; date: 12 January 2023) prior to the start of the study, before the enrolment of the first patient.

### 2.2. Selection of Participants

Consecutive outpatients with a confirmed UC diagnosis, treated in the Clinic for Gastroenterology and Hepatology during the study period, were evaluated for inclusion in the study. UC was diagnosed according to clinical, laboratory, endoscopic and pathohistological findings [30].

The inclusion criteria were age > 18 years, provided signed informed consent, completed all questionnaires, and had a confirmed UC diagnosis.

The exclusion criteria were refusal to participate in study, inability to complete questionnaires due to cognitive deficit or language issues, or had an unconfirmed UC diagnosis.

### 2.3. Instruments

The collection of data on the socio-demographic and clinical characteristics of the participants (HRQOL, symptoms of anxiety, depression and alexithymia) was carried out using questionnaires. The questionnaires used in this study were as follows: a socio-demographic questionnaire, the Short Inflammatory Bowel Disease Questionnaire (SIBDQ), the Patient Health Questionnaire-9 (PHQ-9), General Anxiety Disorder-7 (GAD-7) and the Toronto Alexithymia Scale (TAS-20). The licensed Serbian translation of the SIBDQ was used. The PHQ-9, GAD-7 and TAS-20 were translated and validated in the Serbian language. All the questionnaires contain questions that are closed-ended. The questionnaires were completed by the patients in writing.

The patients with psychological scores suggesting depression and anxiety were advised to consult with a mental health professional.

### 2.4. Questionnaire on Socio-Demographic Characteristics

A standardized questionnaire was used to collect the following information: name and surname, sex, year of birth, education level, occupation, marital and employment statuses, income level, smoking status and alcohol consumption level. This section was completed by the patient. The next section of this questionnaire, which describes the clinical characteristics of the disease, was completed by the treating physician and was used to collect the following information: extent of the disease according to the Montreal classification, year of diagnosis, laboratory parameters (CRP, Hgb, Fe, ferritin, and fecal calprotectin), presence of extraintestinal manifestations, surgeries, comorbidities, allergies, presence of clinical (S0 according to Montreal classification) and laboratory remission and the ongoing therapy for UC [31]. Data on the clinical parameters of the patients with an IBD were obtained from their medical records.

### 2.5. Laboratory Analyses

Laboratory analyses were conducted within a few days of completing the questionnaire. The presence of laboratory remission was defined as unaltered levels of biomarkers, CRP and fecal calprotectin, and the absence of disease-related anemia.

### 2.6. Quality of Life Assessment (SIBDQ)

The SIBDQ is a questionnaire that is used to specifically assess the HRQOL of patients with an IBD [32]. It contains 10 questions covering 4 domains of quality of life: bowel, systemic, social and emotional. Each question offers 7 response options on a Likert scale, ranging from 1 (a severe problem) to 7 (not a problem at all), giving a total SIBDQ score ranging from 10 (poor HRQOL) to 70 (optimal HRQOL). There are no validated cut-offs for the different dimensions’ scores. A SIBDQ score below 50 was considered as poor HRQOL.

### 2.7. Depression Symptom Score Assessment (PHQ-9)

The PHQ-9 is a self-assessment questionnaire that diagnoses and determines the severity of depression based on 9 major depressive criteria. The responses are scored 0 (never) to 3 (every day) [33]. The score ranges from 0 to 27, with a higher score indicating a greater degree of depression. Scores of 5, 10, 15, and 20 represent the cut-offs for mild, moderate, moderately severe, and severe depression, respectively. A PHQ-9 score > 9 had a sensitivity of 88% and a specificity of 88% for major depression compared with a structured psychiatric interview as the diagnostic gold standard [33].

### 2.8. Anxiety Symptom Score Assessment (GAD-7)

The GAD-7 is a self-assessment questionnaire for the intensity of symptoms of generalized anxiety disorder [34]. It consists of seven statements related to the core symptoms of the disorder (e.g., “I worry too much about different things”). Respondents are asked to indicate how often they experienced any of the listed symptoms over the previous two weeks. The responses are given on a Likert scale from 0 (“not at all”) to 3 (“nearly every day”). The total score from the questionnaire indicates the intensity of the symptoms of generalized anxiety disorder. The results are categorized into four levels based on the intensity of anxiety symptoms: a score of 0 to 4 indicates minimal anxiety, 5 to 9 indicates mild anxiety, 10 to 14 indicates moderate anxiety, and 15 to 21 indicates a high anxiety symptom intensity. Using the threshold score of 10, the GAD-7 sensitivity is 89% and specificity is 82% compared to a structured psychiatric interview as the diagnostic gold standard [35]. 

### 2.9. Alexithymia Assessment (TAS-20)

The TAS-20 has 20 questions divided into 3 domains: difficulties in identifying emotions, difficulties in expressing emotions and externally oriented thinking (the tendency to focus attention outward) [36]. A score of up to 51 indicates no alexithymia, 52–60 indicates possible alexithymia, while a score above 60 indicates its presence. The test is devised not only to calculate total scores, but also to associate a score with each one of the aforementioned three dimensions.

### 2.10. Statistical Processing and Data Analysis

The data were analyzed using IBM SPSS 17.0 software package for Windows. Descriptive statistical methods were used to describe the socio-demographic characteristics of the patients. Parametric data are expressed as means and standard deviations, while non-parametric data are expressed as medians. The prevalences of anxiety, depression and alexithymia were calculated as the proportion of individuals with an IBD who have specific mental disorders out of all the individuals in the sample. Hierarchical regression analysis was performed to identify the factors associated with HRQOL in UC. The possible confounding variables identified in our study included age, sex, presence of comorbidities, previously diagnosed mental disorders and effects of medication, particularly corticosteroids. The effects of possible covariates were excluded using hierarchical regression analysis. The dependent variable was HRQOL, expressed as the SIBDQ score. Model 1 includes sex and age as independent variables or predictors. Model 2 adds disease duration, remission status, extraintestinal manifestations and advanced therapy treatment. Model 3 incorporates measures of depression, anxiety and alexithymia (PHQ-9, GAD-7 and TAS-20 total scores, respectively). A *p* value < 0.05 was considered statistically significant. 

## 3. Results

### 3.1. Demographic Characteristics of Patients

This cross-sectional study included 248 patients with UC, of whom, 21 (8.5%) had proctitis, 63 (25.4%) had left-sided UC and 164 (66.1%) had extensive colitis. The demographic characteristics of the participants are described in Table 1. The mean age of our patients was 45 ± 15 years, with the majority having a secondary school-level education(52.4%) and were employed (64.0%), married (52%) and had a monthly income between RSD 50,000 and 100,000 (51.9%). In terms of life habits, 62.9% were non-smokers and 50% had never consumed alcohol.

### 3.2. Clinical Characteristics of Patients with Ulcerative Colitis

The clinical characteristics of the study population are described in Table 2. The mean age at UC diagnosis was 36 ± 14 years. The average disease duration was 9.3 ± 7.4 years. The average CRP value was 1.7 g/L (range 0–298.7). At the time the study was conducted, 95.6% of the patients were in clinical and laboratory remission. Extraintestinal manifestations were present in 22.6% of the patients, while 23.4% had a comorbidity. When it came to treatment, 49.6% of the participants were on mesalazine, 4.0% received corticosteroid treatment in the year before they enrolled in the study, 52% were on immunosuppressants and 79.8% were treated with advanced therapies (biologic drugs or small molecules). None of the patients were using corticosteroid therapy at the time the study was conducted.

### 3.3. HRQOL and Prevalence of Depression, Anxiety and Alexithymia

The patients’ scores on the PHQ-9, GAD-7, TAS-20 and SIBDQ are shown in Table 3. The average SIBDQ score was 56.5 ± 10.9 (range 16–70). Poor HRQOL (SIBDQ < 50) was observed in 61 participants (24.6%). The average SIBDQ score in the UC patients in remission was 56.9, while those with active disease had an average SIBDQ score of 46.8.

The prevalence of depression, assessed using the PHQ-9 symptom score and a cut-off of 5 (including patients with mild depression), was 44.7%. If a PHQ-9 threshold score of 10 (excluding patients with mild depression) was used, the prevalence of depression was 14.5%.

Based on GAD-7, the prevalence of anxiety was 34.3%. A total of 30.2% of the participants had possible alexithymia, and 13.7% had alexithymia.

**Figure 1:**
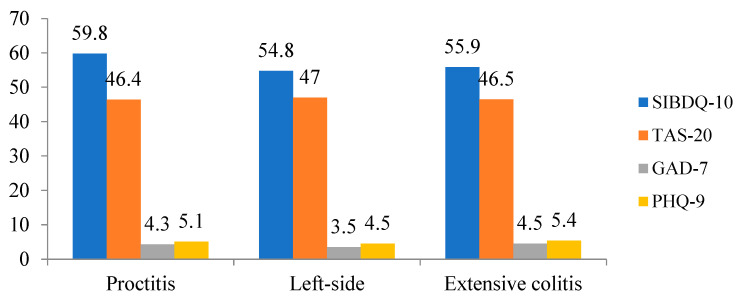
SIBDQ, TAS-20, GAD-7 and PHQ-9 score distributions according to UC localization.

Figure 1 shows the SIBDQ, TAS-20, GAD-7 and PHQ-9 score distributions according to the localization of the UC. None of the scales reached statistical significance (for SIBDQ: F = 1.668, *p* = 0.191; for TAS-20: F = 0.041, *p* = 0.960; for GAD-7: F = 0.928, *p* = 0.397; for PHQ-9: F = 0.897, *p* = 0.409). When the participants were compared in terms of treatment with advanced therapy, none of the scores differed (PHQ-9: *p* = 0.565; GAD-7: *p* = 0.891; TAS-20: *p* = 0.874; SIBDQ: *p* = 0.117).

**Figure 2:**
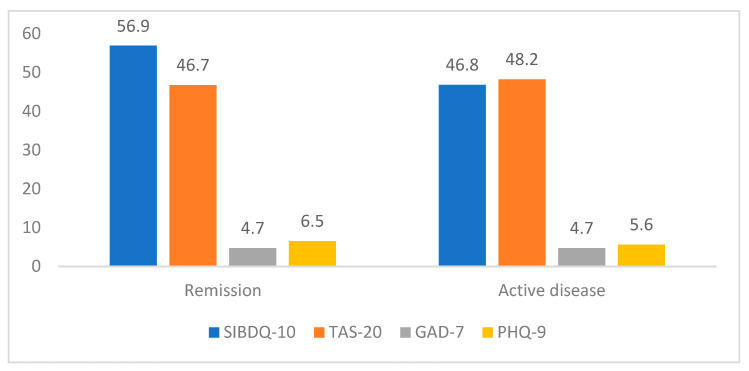
SIBDQ, TAS-20, GAD-7 and PHQ-9 score distributions between UC patients in remission and patients with active disease.

Figure 2 shows the SIBDQ, TAS-20, GAD-7 and PHQ-9 score distributions between the patients in remission and the patients with active UC. The remission group had a statistically significant higher SIBDQ score (F = 0.667, *p* = 0.002). The scores for the mental health scales of the patients with active disease and those in remission did not differ significantly (for TAS-20: F = 0.001, *p* = 0.550; for GAD-7: F = 0.330, *p* = 0.972; for PHQ-9: F = 1.237, *p* = 0.294).

Table 4 presents the SIBDQ score distribution according to the level of depression, anxiety and alexithymia. All the scale scores including the PHQ-9, GAD-7 and TAS-20 scores were associated with the HRQOL of the UC patients, as measured by the SIBDQ score. The strongest correlation was observed between depression severity, measured by the PHQ-9 score and SIBDQ score (r = −0.584, *p* < 0.001), which was negative, indicating that a higher depression score among the UC patients correlated with worse HRQOL. Similarly, anxiety level moderately and negatively correlated with SIBDQ scores (r = −0.398, *p* < 0.001), indicating an association between a higher anxiety level and impaired HRQOL in UC patients. The relationship between the TAS-20 and SIBDQ scores was also negative but it was weak-to-moderate in strength (r = −0.339, *p* < 0.001), showing that an increase in the alexithymia score significantly deteriorates the HRQOL of UC patients.

### 3.4. Regression Analysis of Factors Associated with SIBDQ Score

In Table 5, the results of the hierarchical regression analysis of factors influencing the SIBDQ score in the UC patients is shown. In model 1 and model 2, the effect of sex was positive, but very small and non-significant (β = 0.03 and 0.04, respectively). In model 3, the relationship became slightly negative (β = −0.06) but remained non-significant. Across all the models, age had a minimal effect, with consistently non-significant β values (close to 0). In model 2 and model 3, disease duration showed negligible effects (β = −0.01 and 0.03, respectively), indicating no meaningful relationship. In model 2, remission had a significant positive effect (β = 0.20, *p* < 0.01), indicating that the patients in remission tended to have higher SIBDQ scores. In model 3, this remained significant, but the effect slightly decreased in strength (β = 0.17, *p* < 0.01). In model 2, extraintestinal manifestations had a significant negative effect (β = −0.13, *p* < 0.05), indicating worse scores for those with extraintestinal manifestations. In model 3, this effect became non-significant (β = −0.02). Advanced therapy treatment, in model 2 and model 3, had no significant effect (β close to 0). The PHQ-9 score, in model 3, had a strong negative effect (β = −0.62, *p* < 0.01), which indicates that higher depression scores were significantly associated with worse outcome scores. The GAD-7 score, in model 3, had no significant effect (β = −0.06), suggesting that anxiety had little additional influence when controlling for other variables. The TAS-20 score, in model 3, had no significant effect (β = 0.01), indicating alexithymia was not a key predictor in this model. Remission status was consistently associated with higher scores, indicating better outcomes for patients in remission. Extraintestinal manifestations negatively affected the SIBDQ scores, but this effect diminished after controlling for psychological variables. The depression symptom (PHQ-9) score was the strongest predictor in model 3. The anxiety symptom (GAD-7) score and alexithymia (TAS-20) score did not contribute much when depression was included in the model. Overall, the variables in the first model (age and sex) explained 0.4% of the variance in the SIBDQ scores (F for change in R^2^ = 0.525, *p* > 0.05). Adding remission status, presence of extraintestinal manifestations and treatment with advanced therapies explained another 5.3% of the variance (F for change in R^2^ = 2.904, *p* < 0.05). Finally, including the PHQ-9, GAD-7 and TAS-20 total scores resulted in 40.6% of the variance being explained (F for change in R^2^ = 25.776, *p* < 0.01). This means that all the variables in the three models explained a total of 46.3% of the difference in SIBDQ scores in the patients with UC.

## 4. Discussion

### 4.1. Demographic and Clinical Data

This cross-sectional study included 248 patients diagnosed with UC, of whom, 21 (8.5%) had proctitis, 63 (25.4%) had left-sided UC and 164 (66.1%) had extensive colitis. The mean age of our patients was 45 ± 15 years. The mean age at UC diagnosis was 36 ± 14 years, which is consistent with previously published results [37]. The average disease duration was 9.3 ± 7.4 years (range 0–49 years). The gender distribution was equal, with a slight female predominance since we included 131 (52.8%) females and 117 (47.2%) males, which is in agreement with the majority of studies describing an equal distribution of UC between genders [37].

Age and gender did not impact the HRQOL in our patients, which was shown by the regression analysis. The majority of the UC patients in our study had a secondary-level education (52.4%), were married (52%) and employed (64.0%), with a moderate monthly income (51.9%). The Global Burden of Disease study suggested higher IBD rates in individuals with a higher socioeconomic status, while our findings revealed that only 8.2% patients had a high monthly income [38].

In terms of life habits, 62.9% of the patients had never smoked, 20% were active smokers and 16.9% were ex-smokers, which is consistent with the previously reported possible protective role of smoking in UC patients [37]. Nevertheless, the data from a recent study revealed that current smokers had a 2.3-fold higher risk of UC in prospective analyses vs. those who have never smoked [39]. The smoking status after diagnosis, according to published data, does not affect the disease course or clinical outcomes [40].

At the time the study was conducted, 95.6% of the participants were in clinical and laboratory remission, resulting in normal average values for their laboratory parameters. Extraintestinal manifestations were present in 22.6% patients, while 23.4% had a comorbidity. Only one (0.4%) participant had a previously diagnosed depressive disorder.

In the hierarchical regression analysis, extraintestinal manifestations negatively affected the SIBDQ score, but this effect disappeared after controlling for psychological variables.

Half of the participants were treated with mesalazine and 52% were on immunosuppressants. Ten patients (4%) were treated with corticosteroids in the year before the study, but none of the patients were taking corticosteroids during the study period. The majority of patients (79.8%) were treated with advanced therapies (biologic drugs or small molecules) since our study was conducted at a tertiary referral center. The high percentage of patients using advanced therapy may not be reflective of a broader UC population, but it includes UC patients with severe form of disease in whom low HRQOL and psychological disorders are to be expected.

### 4.2. Health-Related Quality of Life

The average SIBDQ score was 56.5 ± 10.9. Poor HRQOL (SIBDQ < 50) was observed in 61 of the UC patients (24.6%). The patients in remission, as expected, had better SIBDQ scores compared to those experiencing active symptoms (56.9 vs. 46.8, respectively, *p* = 0.002). The results of the hierarchical regression analysis showed that remission of disease and a higher depression score were the main factors influencing HRQOL in the UC patients.

In previously published studies, disease activity was found to be the most important predictor of HRQOL [41,42]. A prospective observational study on 115 patients with an IBD found that clinical remission normalized the HRQOL in 82% of the patients with UC, which, importantly, was not related to the type of treatment [43]. In a group of IBD patients in remission, the low HRQOL was significantly associated with surgery, disease duration, sleep disturbance, anxiety/depression and high illness perception [44]. Anxiety, depression, fatigue and IBS symptoms were all independently associated with a lower HRQOL in patients with an inactive IBD [45]. Psychological symptoms of emotional disorders appear to be associated with a lower HRQOL in IBD patients [46].

We found no differences in HRQOL regarding the use of advanced therapies. Among UC patients in a real-world setting, the patients on advanced therapies were less likely to be in remission, and had more moderate-to-severe disease and worse patient reported outcome (PROs) than patients on mesalazine therapy [47]. The results of a meta-analysis suggested that biologics have the potential to improve the HRQOL of UC patients [48]. High-quality evidence suggests that infliximab provides a clinically meaningful improvement in HRQOL in UC patients receiving induction therapy. Moderate-quality evidence suggests that vedolizumab provides a clinically meaningful improvement in HRQOL in UC patients receiving maintenance therapy. Biological therapies have shown improvement in the HRQOL of patients with ulcerative colitis during the induction phase, with benefits that are maintained during maintenance treatment [48,49]. Studies have indicated that small molecules can improve HRQOL [49,50].

### 4.3. Depression, Anxiety and Alexithymia in UC Patients

The instruments used for the assessment of HRQOL, depression, anxiety and alexithymia in UC vary among studies [51]. In our study, the prevalence of depression, anxiety and alexithymia was 44.7%, 34.3% and 30.2%, respectively, which is consistent with previously published data [8,14,15,52]. There were no differences in the PHQ-9, GAD-7 and TAS-20 scores in relation to remission status (*p* > 0.05).

It was previously reported that during the active phase of the disease, one in two patients is either anxious or depressed [14]. Prior investigations revealed that in patients newly diagnosed with moderate-to-severe UC, the prevalence of depression was 41.8% and that of anxiety was 33.7% in the first four weeks after diagnosis [8]. A Chinese cross-sectional study showed that 58% of patients with active UC had depression, while 50% had anxiety [52].

A retrospective study of 327 outpatient IBD patients showed that the prevalence of anxiety was 21.2% and that of depression was 25.8% [53]. In that study, the presence of depression and/or anxiety was determined using the PHQ-9 and GAD-7 self-report questionnaires or by diagnosis through a psychiatric interview.

A prospective cohort study from Manitoba, which included 363 participants, found that the prevalence of depression was twice as high in the IBD group compared to the control group, with 27.2% of the IBD patients and 12.3% of the controls being affected [9]. The IBD subtype (CD or UC) was unrelated to a psychiatric diagnosis. The strength of the Manitoba study is that they used a psychiatric interview, the composite international diagnostic interview, based on diagnostic criteria from the *Diagnostic and Statistical Manual of Mental Disorders (DSM-IV)* published by the American Psychiatric Association, which is the gold standard for diagnosing depression [54].

In one study, 33% of IBD patients had symptoms of anxiety and 16.5% had symptoms of depression [55]. They found that exposure to more than one biologic therapy, fatigue and symptoms of anxiety were associated with a lower HRQOL in the multivariate analysis.

A review of seven studies found that the incidence of depression was 3.6–26.9/1000 person-years in IBD patients, compared to 2.5–12.2/1000 person-years in the reference population [15]. The incidence of anxiety in the IBD patients was 4.0–25.0/1000 person-years, while in the controls, it was 3.0–16.3/1000 person-years. A meta-analysis of five studies examining the risk of anxiety and depression after an IBD diagnosis showed that IBD patients had an increased risk of anxiety (HR: 1.48; 95% CI: 1.29–1.70) and depression (HR: 1.55; 95% CI: 1.35–1.78) [15].

We did not find an association between mental disorders and the use of biological therapy, which is consistent with the results of a study with a similar methodology [23].

The prevalence of psychological disturbances in IBDs varies across studies, owing to heterogeneity in the study populations, time of assessment (whether it is across the lifespan or over a single period of life, usually the first year after the IBD diagnosis) or assessment tools for depression and anxiety.

The prevalence of alexithymia in our study was 30.2%, which is consistent with the literature data [27,56,57]. The mean TAS-20 score was 46.7 ± 12.3.

A total of 41 patients (16.5%) had possible alexithymia and 34 patients (13.7%) had a TAS-20 score >61, indicating they had alexithymia. In one systematic review, the mean alexithymia score ranged from 39 to 53.2, thus mostly falling in the normal range and indicating no alexithymia and the authors concluded that IBD patients cannot be considered alexithymic to a clinically significant extent [27]. They are more alexithymic compared to healthy participants, similar to patients with irritable bowel syndrome, and less alexithymic than patients with major depressive disorder. In the aforementioned study, there was an association between the level of alexithymia and other relevant psychological variables, such as anxiety and depression, but that was not confirmed in our study [27].

### 4.4. Influence of Depression, Anxiety and Alexithymia on HRQOL

A higher depression symptom score was one of the main factors influencing HRQOL in the UC patients. Our results are consistent with the results in the literature [8,44,58]. A Korean nationwide prospective cohort study highlighted that the presence of either significant anxiety or depression had a strong negative impact on both disease-specific and generic HRQOL [8]. The prospective study showed that anxiety and/or depression, particularly anxiety, present at the time of UC diagnosis did not influence long-term clinical outcomes (remission rates, response rates or hospitalization rates), but they persistently impaired HRQOL [7]. In one cross-sectional study, a low HRQOL, measured by the SIBDQ score, was associated with UC but not CD, IBD surgery, disease duration, sleep disturbance or anxiety/depression [44].

### 4.5. Other Factors Influencing HRQOL—Possible Role of Gut–Brain Axis and Genetic Factors?

The absence of differences in the PHQ-9 and GAD-7 scores of patients in remission and those with active disease suggests that psychological disorders are not solely caused by the presence of an IBD. Anxiety and depression persisted in the UC patients even during remission.

The gut–brain–microbiota axis establishes a connection between the brain and the vast community of bacteria in the intestinal tract [18]. Recent studies have shown that the gut microbiome influences brain processes [19]. The ability of the gut microbiota to affect central nervous system (CNS) activities is mainly mediated trough neurological (autonomic nervous system), hormonal (hypothalamus–pituitary–adrenal axis) and immunological (cytokine and chemokine) processes, all of which are interrelated [59]. The gut microbiome synthesizes various molecules that can impact brain function, including neurotransmitters (dopamine, serotonin, GABA, short chain fatty acids (SCFAs) and tryptophan). Psychological disorders are related to reduced gut microbiota richness and diversity. Compared to UC patients without depression and or anxiety, those with these conditions exhibit lower microbial diversity, a decrease in anti-inflammatory SCFA (butyrate)-producing bacteria, an increase in lactic acid-producing bacteria and higher levels of bacteria associated with glutamate and GABA metabolism [52,60,61].

Recent Mendelian randomization studies suggested a genetic predisposition to depression increases the risk of IBDs (both CD and UC). Also, the authors suggested that genetic susceptibility to UC is associated with anxiety, but not vice versa [62,63].

### 4.6. Strengths and Limitations of the Study

The strengths of our study are as follows:

- The study focused on an important clinical issue: the psychological burden of UC and its effects on HRQOL.
- We simultaneously investigated HRQOL and anxiety/depression status, along with the clinical and demographic characteristics of a large group of UC patients.

The limitations of the study are as follows:

- The institution where the study was conducted is a tertiary center and mainly treats patients with more severe and complex disease behaviors. A multicenter study, with a larger number of patients and more patients on conventional therapy, would provide a more accurate assessment of HRQOL, as well as the prevalence of anxiety, depression and alexithymia in the population of patients with UC as a whole.
- A cross-sectional study design cannot reveal the direction of causal relationships between psychological disorders, UC and HRQOL and the long-term impact of psychological factors on disease progression and HRQOL.
- In this study, self-administered questionnaires were used, which were completed by the participant on their symptoms of anxiety, depression, and alexithymia at the time of assessment.
- Questionnaires are not used for diagnosing psychiatric disorders, but they can be used for screening and identifying patients who require further psychiatric evaluation. The gold standard for diagnosing these disorders remains a psychiatric interview.

## 5. Conclusions

The prevalence of depression, anxiety and alexithymia in UC patients was high, even if the disease was in remission. Remission of disease and a high depression score were the main factors related to HRQOL. Depression adversely affected HRQOL in the UC patients, highlighting the need for psychological screening and mental health support for all UC patients.

We strongly recommend, based on our study results, routine mental health screening for all UC patients and referrals to mental health specialists if needed.

## Figures and Tables

**Table 1:** Demographic characteristics of patients with ulcerative colitis.

Variable	Frequency (%)
Sex (%) Male Female	117 (47.2) 131 (52.8)
Age (years, mean ± SD)	45 ± 15
Educational level (%) Primary school Secondary school College Faculty	12 (4.8) 130 (52.4) 31 (12.5) 75 (30.2)
Working status (%) Permanent job Temporary job Unemployed Retired	137 (55.5) 21 (8.5) 45 (18.2) 44 (17.8)
Marital status (%) Married Extramarital union Widowed Divorced Single	129 (52.0) 16 (6.5) 14 (5.6) 21 (8.5) 68 (27.4)
Income level (%) <RSD 50,000 RSD 50–100,000 >RSD 100,000	97 (39.9) 126 (51.9) 20 (8.2)
Smoking status (%) Non-smoker Smoker Ex-smoker	156 (62.9) 50 (20.2) 42 (16.9)
Alcohol consumption (%) Never Up to 1 glass/day On occasion	124 (50.0) 4 (1.6) 120 (48.4)

SD—standard deviation; RSD—Serbian dinar.

**Table 2:** Clinical characteristics of patients.

Variable	Value	Accepted Normal Range
Age at diagnosis (years)	36 ± 14	
Age at diagnosis (%) <16 years of age 17–40 years of age >40 years of age	12 (4.8) 161 (64.9) 75 (30.2)	
Disease duration (years)	9.3 ± 7.4	
CRP (g/L) (median, range)	1.7 (0–298.7)	0–5
Hemoglobin (g/L)	139.7 ± 17.3	120–160
Iron (μmolL)	14.9 ± 7.6	11–32
Ferritin (μgL) (median, range)	48.5 (0.9–580)	30–100
Calprotectin (μg/g) (median, range)	81.45 (0–2000)	0–100
**In remission at the time of the study (%)** Yes No	237 (95.6) 11 (4.4)	
**Extraintestinal manifestations of disease (%)** Yes No	56 (22.6) 192 (77.4)	
**Presence of comorbidity (%)** Yes No	58 (23.4) 190 (76.6)	
**Prior mental disorder (%)** Yes No	1 (0.4) 247 (99.6)	
**IBD therapy (%)** ***Mesalazine*** ***Corticosteroid treatment in last year*** ***Immunosuppressants*** Azathioprine Methotrexate ***Advanced therapies*** Infliximab Infliximab biosimilar Adalimumab Adalimumab biosimilar Vedolizumab Tofacitinib	123 (49.6) 10 (4.0) 129 (52.0) 115 14 198 (79.8) 23 83 34 2 52 4	

**Table 3:** SIBDQ, PHQ-9, GAD-7 and TAS-20 scores in patients with UC.

Variable	Value	Score Range
SIBDQ (range)	56.5 ± 10.9 (16–70)	10–70
SIBDQ categories (%) Poor HRQOL Optimal HRQOL	61 (24.6) 187 (75.4)	SIBDQ < 50 SIBDQ ≥ 50
PHQ-9 total score (range)	5.2 ± 4.5 (0–27)	0–27
PHQ-9 categories (%) None–minimal depression Mild depression Moderate depression Moderately severe depression Severe depression	137 (55.2) 75 (30.2) 25 (10.1) 9 (3.6) 2 (0.8)	0–4 5–9 10–14 15–19 20–27
GAD-7 total score (range)	4.3 ± 4.7 (0–21)	0–21
GAD-7 categories (%) Minimal anxiety Mild anxiety Moderate anxiety Severe anxiety	163 (65.7) 51 (20.6) 21 (8.5) 13 (5.2)	0–4 5–9 10–14 15–21
TAS-20 total score (range)	46.7 ± 12.3 (20–94)	20–100
TAS-20 categories (%) No alexithymia Possible alexithymia Alexithymia present	173 (69.8) 41 (16.5) 34 (13.7)	20–51 52–60 61–100

**Table 4:** SIBDQ score values according to level of depression, anxiety and alexithymia.

Variable	Value	
	Score	Correlation with SIBDQ
**PHQ-9 categories** None–minimal depression Mild depression Moderate depression Moderate-severe depression Severe depression	61.6 ± 7.2 53.7 ± 9.4 46.1 ± 11.6 37.1 ± 8.5 29.0 ± 5.7	r = −0.584 *p* < 0.001
**GAD-7 categories** Minimal anxiety Mild anxiety Moderate anxiety Severe anxiety	59.4 ± 8.7 54.8 ± 11.0 50.2 ± 9.4 36.4 ± 11.3	r = −0.398 *p* < 0.001
**TAS-20 categories** No alexithymia Possible alexithymia Alexithymia present	58.6 ± 10.0 54.2 ± 11.0 48.2 ± 10.8	r = −0.339 *p* < 0.001

**Table 5:** Factors associated with SIBDQ score in UC patients—hierarchical regression analysis.

Variable	Model 1			Model 2			Model 3		
	B	SE (B)	β	B	SE (B)	β	B	SE (B)	β
Sex	0.64	1.39	0.03	0.81	1.37	0.04	−1.24	1.05	−0.06
Age	0.04	0.05	0.06	0.04	0.05	0.06	−0.01	0.04	−0.01
Disease duration				−0.01	0.10	−0.01	0.04	0.08	0.03
Remission yes vs. no				9.62	3.32	0.20	9.10	2.53	0.17
Extraintestinal manifestations yes vs. no				−3.28	1.63	−0.13	−0.54	1.26	−0.02
Advanced therapy treatment yes vs. no				−0.55	1.86	−0.02	0.65	1.42	0.02
PHQ-9 total score							−1.47	0.19	−0.62
GAD-7 total score							−0.13	0.18	−0.06
TAS-20 total score							0.01	0.05	0.01
*R* ^2^	0.004			0.057			0.463		
*F* for change in *R*^2^	0.525			2.904			25.776		

## Data Availability

The raw data supporting the conclusions of this article will be made available by the authors on request.

## Abbreviations

The following abbreviations are used in this manuscript:

IBD	Inflammatory bowel disease
UC	Ulcerative colitis
CD	Crohn’s disease
SIBDQ	Short Inflammatory Bowel Disease Questionnaire
PHQ- 9	Patient Health Questionnaire-9
GAD-7	General Anxiety Disorder-7
TAS-20	Toronto Alexithymia Scale
